# Understanding the implementation of interventions to improve the management of chronic kidney disease in primary care: a rapid realist review

**DOI:** 10.1186/s13012-016-0413-7

**Published:** 2016-04-04

**Authors:** Jung Yin Tsang, Tom Blakeman, Janet Hegarty, John Humphreys, Gill Harvey

**Affiliations:** 1 NIHR Collaboration for Leadership in Applied Health Research (CLAHRC) Greater Manchester (GM), Centre for Primary Care, Institute of Population Health, University of Manchester, Manchester, M13 9PL UK; 2 Renal Department, Salford Royal NHS Foundation Trust, Stott Lane, Salford, M6 8HD UK; 3 Alliance Manchester Business School, University of Manchester, Booth Street West, Manchester, M15 6PB UK; 4 School of Nursing, University of Adelaide, Eleanor Harrald Building, Frome Road, Adelaide, SA5005 Australia

**Keywords:** Chronic kidney disease, Primary care, Family practice, Implementation, Interventions, Quality improvement, Rapid realist review, Normalisation Process Theory

## Abstract

**Background:**

Chronic kidney disease (CKD) is common and a significant marker of morbidity and mortality. Its management in primary care is essential for maintenance of cardiovascular health, avoidance of acute kidney injury (AKI) and delay in progression to end-stage renal disease. Although many guidelines and interventions have been established, there is global evidence of an implementation gap, including variable identification rates and low patient communication and awareness. The objective of this study is to understand the factors enabling and constraining the implementation of CKD interventions in primary care.

**Methods:**

A rapid realist review was conducted that involved a primary literature search of three databases to identify existing CKD interventions in primary care between the years 2000 and 2014. A secondary search was performed as an iterative process and included bibliographic and grey literature searches of reference lists, authors and research groups. A systematic approach to data extraction using Normalisation Process Theory (NPT) illuminated key mechanisms and contextual factors that affected implementation.

**Results:**

Our primary search returned 710 articles that were narrowed down to 18 relevant CKD interventions in primary care. Our findings suggested that effective management of resources (encompassing many types) was a significant contextual factor enabling or constraining the functioning of mechanisms. Three key intervention features were identified from the many that contributed to successful implementation. Firstly, it was important to frame CKD interventions appropriately, such as within the context of cardiovascular health and diabetes. This enabled buy-in and facilitated an understanding of the significance of CKD and the need for intervention. Secondly, interventions that were compatible with existing practices or patients’ everyday lives were readily accepted. In contrast, new systems that could not be integrated were abandoned as they were viewed as inconvenient, generating more work. Thirdly, ownership of the feedback process allowed users to make individualised improvements to the intervention to suit their needs.

**Conclusions:**

Our rapid realist review identified mechanisms that need to be considered in order to optimise the implementation of interventions to improve the management of CKD in primary care. Further research into the factors that enable prolonged sustainability and cost-effectiveness is required for efficient resource utilisation.

**Electronic supplementary material:**

The online version of this article (doi:10.1186/s13012-016-0413-7) contains supplementary material, which is available to authorized users.

## Background

Chronic kidney disease (CKD) is an increasingly common condition, with a global prevalence estimated at 8–16 % [1]. It is defined as reduced kidney function, demonstrated by decreased estimated glomerular filtration rate (eGFR), or evidence of kidney damage, such as increased excretion of urinary albumin [2]. CKD is rarely diagnosed in isolation and is associated with considerable co-morbidity, especially in the elderly population [3, 4]. Ninety-seven percent of patients with moderate to severe CKD have mostly asymptomatic stage 3 disease, but even this stage of CKD bears a two- to fourfold rise in cardiovascular disease risk and a significant increase in all-cause mortality [5, 6]. Only a small proportion of patients progress to end-stage renal disease (i.e. stage 5 disease), but this requires costly treatments and is associated with substantial morbidity and mortality [5]. Furthermore, evidence shows that CKD is a significant risk factor for patients developing acute kidney injury (AKI) [7]. AKI causes an increase in cost, length of stay and readmission rates to hospitals as well as raised short- and long-term mortality rates [7, 8].

Several international guidelines exist to direct the treatment of CKD, with an emphasis on effective management of early stage disease [1, 3]. Hence, the primary care management of early stage CKD is essential, harbouring the potential to prevent complications and improve health outcomes [3, 9]. To support the implementation of these guidelines from a UK perspective, CKD was included in the national ‘pay for performance’ scheme for primary care from 2006, called the Quality and Outcomes Framework (QOF) [10]. This scheme has undergone several modifications but included financial incentives for attainments such as the recognition of CKD, blood pressure (BP) management and the appropriate use of renin-angiotensin system agents [4, 10].

However, global evidence repeatedly shows that there is an implementation gap between CKD guidelines and clinical practice [11–13]. This includes variable levels of recognition, difficulties in communicating the diagnosis to patients, poor patient awareness and uncertainty surrounding medication and referrals [14, 15]. Studies have highlighted the need to understand the issues surrounding implementation to close the translational gap and inform healthcare professionals on effective intervention design [4, 16, 17].

The purpose of this study was to deepen the understanding of the factors affecting the implementation of CKD interventions in primary care. Guided by the framework of a rapid realist synthesis, the focus was on the following research questions:

1. What mechanisms enabled components of a CKD intervention to be successfully implemented into primary care?
2. What were the underlying contextual conditions that activated these mechanisms?

From this review, the aim was to produce informative results that could be used to inform intervention design and advise policymakers.

## Methods

### Rapid realist review and Normalisation Process Theory

This study has been informed by the principles of a rapid realist review combined with Normalisation Process Theory (NPT). The RAMESES standards were adopted to conduct the review [18].

Realist methodology is a systematic approach designed for analysing complex interventions. It combines both qualitative and quantitative data to perform a multidimensional investigation [19]. It allows a deeper exploration into the underlying mechanisms that lead to a particular outcome, whilst considering specific contextual factors. Compared to a ‘traditional’ realist approach, a rapid realist review is less concerned with theory development and more focussed on explanations [20]. It is a time-responsive method allowing the generation of findings to inform policy and clinical practice.

NPT is a middle range theory that is concerned with the underlying processes surrounding implementation [21]. Its development was grounded in primary care and attempts to take into account complex interplaying factors of work and action rather than attitudes and beliefs. It was designed to identify and understand the processes underpinning care, through which existing interventions had become taken-for-granted or ‘normalised’ [21, 22]. These are described as coherence, cognitive participation, collective action and reflexive monitoring [21]. From a realist perspective, these can be viewed as basic mechanisms through which implementation and therefore normalisation occurs.

### Design

Rapid realist methodology was identified as an appropriate approach to construct an analysis of how complex primary care interventions work for CKD in particular situations (i.e. ‘what works, for whom and in what circumstances’) [19]. From the perspective of a realist approach, we concentrated on identifying the underlying mechanisms activated by interventions and the contextual features required to produce successful outcomes in terms of implementation. As heterogeneous data from both quantitative and qualitative studies were included, the review set out to look for definitions for the outcome of ‘successful implementation’ within the data. The focus and purpose of the review, along with the review questions, were refined by the research team from the initial scope. This allowed us to modify our application of NPT as a middle range theory to illuminate additional data and theories regarding implementation.

NPT was used as a sensitisation tool to frame both data synthesis and analysis. The approach chosen accepted the body of evidence surrounding the development of NPT and its ‘domains’ as categories of basic mechanisms. Informed by NPT, we started from the proposition that achieving the basic mechanisms of ‘coherence’ (that is, sense-making work), ‘cognitive participation ‘(engagement work), ‘collective action’ (operational work) and ‘reflexive monitoring’ (feedback and quality improvement work) [21] contributed to successful implementation. In doing so, it provided a framework to identify underlying mechanisms or actions within each of these ‘domains’ that affect implementation at the micro-level.

### Search process

**Fig. 1:**
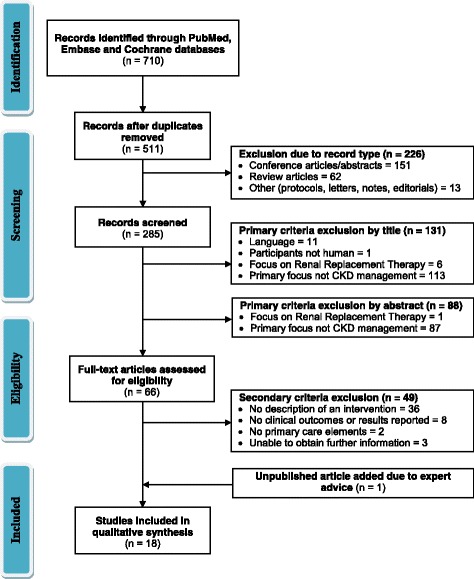
Modified PRISMA flow diagram for the primary literature search

The search process was performed in two stages. The primary search was used to identify published CKD interventions with a strict primary care focus (summarised in Fig. 1). All study designs were included in order to perform an in-depth exploration of the factors surrounding implementation, taking into account that there would be varying strengths of evidence. Seeking to understand the complexities surrounding implementation, both practitioner and patient interventions were included. Members of the review team have also been involved in developing CKD interventions, and hence, recommendations and unpublished reports were also examined. The primary search was performed to identify results from the years 2000 to 2014. The primary and secondary exclusion criteria and search terms are included in Table 1.

**Table 1:** The primary and secondary exclusion criteria and search terms for the primary search

Primary exclusion criteria
• Studies not written in English
• Studies that include participants which are not human
• Studies where the primary focus was not on the management of kidney disease
• Studies which focussed on participants on renal replacement therapy
• Studies which were letters, notes, conference abstracts or reviews only
Secondary exclusion criteria
• Studies where there was no description of any intervention
• Studies that did not report any clinical outcomes or results
• Studies where there were no primary care elements
• Unable to obtain further information to make assessment
Search terms used
((Chronic Kidney Disease or CKD) and (intervention or interventions or tool or tools or strategy or strategies or project or projects or model or models or scheme or schemes or quality improvement or quality improvements) and (Primary care or family practice or general practice))

The secondary search was an iterative process that was performed throughout the project. From the published interventions identified in the primary search, each study was then isolated and the search was expanded to gain additional insight. This entailed

1. Searches of relevant articles in the reference list;
2. Searches of the author on PubMed, Cochrane and Embase;
3. Searches of the author and research group on Google, to identify relevant grey literature.

### Selection, appraisal and extraction

Data from both primary and secondary studies were selected and appraised by assessing their relevance to the research question, transferability of results and the appropriateness of data collection and analysis processes. A data extraction tool (see Additional file 1) was developed in September 2014 by JT and modified after consultation with the other authors. This consisted of three parts to explore the factors surrounding implementation using a realist perspective. Using NPT, Murray et al. [21] further published a series of questions to guide the evaluation of factors affecting the implementation of an intervention on a more practical level. This was included within our extraction tool as a means of data sensitisation to delve deeper into the issues surrounding implementation.

Data extracted included

1. Background information regarding the intervention, such as the setting and demographics to outline possible contextual factors;
2. The underpinning theories or key workings that contributed to the design and functioning of an intervention including information within NPT domains—to identify underlying mechanisms;
3. Information and evidence suggestive of the successes or failures of different aspects of an intervention.

### Analysis and synthesis processes

Data was extracted by JT, and weekly data sessions were held between two authors (JT and TB) to critically appraise, analyse and synthesise the data. After each data session, key themes, contexts and mechanisms were summarised and their relationships elicited. NPT was then used to further evaluate the strengths and weaknesses of each component of an intervention regarding implementation.

Specifically, we attempted to identify mechanisms (individual or collective) that were globally observed across different interventions leading to successful implementation. We also focussed on identifying contextual factors that enabled or inhibited the activation of specific mechanisms. It should be noted that we did not test the effectiveness of interventions identified. Rather, the focus was on different components within each CKD intervention that were successfully or unsuccessfully implemented.

## Results

The review identified 18 full-text articles of existing CKD interventions in primary care. A descriptive overview of the interventions is provided in Table 2. The iterative secondary search further identified 137 records that provided further insight into each of these 18 interventions that were explored in the qualitative synthesis. Further detail of the records identified by the secondary search is provided in Additional file 2, with a summary table in Additional file 3. Parts of both published and grey literature and the qualitative and quantitative aspects that were linked to these papers provided evidence that all interventions included in the review were complex in nature. They required different members of healthcare and non-healthcare staff and organisations to enable the implementation of different components of each intervention. The literature suggested that different aspects from the same intervention had differences in success with some components being well implemented and other features being discarded. Evidence of successful implementation as an outcome included high percentage of uptake, a low dropout rate of the intervention or repeated inferences of acceptability.

**Table 2:** An overview of the 18 CKD interventions for primary care identified from the primary search

	Intervention type	Author (year)	Main intervention description	Other intervention(s)	Sample size	Country	Summary of findings	Other comments
CKD interventions aimed at healthcare professionals	Educational	Cortes-Sanabria et al. (2008) [28]	Intensive weekly teaching sessions to GP (5 h weekly for 6 months)	Validated test at 0 and 6 months to measure competence	94	Mexico	Increased GP competence, led to improved eGFR and BP control, better prescribing	High enrolment rate. 91 % of GPs increased their clinical competence
		Akbari et al. (2004) [26]	2 h of teaching seminars to GPs, with direct access to advice from nephrologist	Automated reporting of eGFR by laboratory	324	Canada	Increased recognition of CKD	Limited data for evaluation, early study
	Practice group meetings	De Lusignan et al. (2013) [34]	Audit-based education (twice yearly feedback about quality and performance compared with peers)	Education, peer support	23,311	UK	Improved BP control and increased use of ACEi. No differences in eGFR	Large study including 93 different practices
		Humphreys et al. (2012) [16]	Three large practice group meetings with local rapid quality improvement cycles (planned and organised by research collaboration)	Implementation team support	5509	UK	CKD recognition, BP control and proteinuria testing all improved	Included 19 different practices
	Multidisciplinary management	Scherpbier et al. (2013) [32]	Shared care between nurse practitioners and GPs (with access to nephrologist or nephrology nurse via digital technology)	Education to both groups	164	Holland	Decreased BP and serum PTH, increased use of ACEi and statins	Limited supporting data for evaluation
		Barrett et al. (2011) [25]	Nurse co-ordinated care (with access to nephrologist)		427	Canada	No difference in rate of decline of eGFR or BP. But an increase in mean eGFR	Most patients ‘extremely satisfied’ with care on questionnaire
		Bayliss et al. (2011) [27]	MDT approach (including nephrologist, pharmacy specialist, diabetes educator, dietitian, social worker, and nephrology nurse)	Components included weekly meetings, contact by telephone or email, individualised plans and patient education	2002	USA	Rate of decline of eGFR improved. No differences in BP, lipids or HbA1C	Limited data to determine which individual components were effectual
		Richards et al. (2008) [33]	Disease management programme (includes patient education, medication review, dietetic advice and social worker)	Desktop guide for clinicians containing clinical management and referral algorithms	483	UK	Improved eGFR, BP and cholesterol.	An extra resource. 85 % enrolment of practices within one area
		Patel et al. (2005) [45]	Pharmacists performing medication reviews		82	USA	Improvement of CKD recognition. No difference in BP, HbA1C or creatinine clearance	99 % of patients had prescription related problems. Only 40.9 % of advice was accepted
	Computer software	Drawz et al. (2012) [36]	Access and training for CKD registries	Educational lecture to both groups, academic detailing	781	USA	Increased PTH measurements, but no difference in BP control	Poor uptake: only 5/37 GPs accessed the registry
		Erler et al. (2012) [35]	Medication alert software with training	1 h education to both groups, patient info leaflets	404	Germany	Improved prescribing	Lack of contextual integration limited its use
		Abdel Kader et al. (2011) [23]	Computer-generated automatic alerts for referral to nephrologist	Two 15 min educational sessions for GPs in both groups	248	USA	No differences in referral to nephrologists or BP control	97 % uptake rate of GPs. No dropouts from study
		Fox et al. (2008) [30]	Computer decision support software generating a recommended to-do list	Ancillary staff + monthly academic detailing	180	USA	Mean eGFR, CKD recognition, anaemia diagnosis all improved	Ancillary staff also did extra work including translating patient guides
	Financial	Karunaratne et al. (2013) [29]	National pay for performance scheme (Quality and Outcomes Framework)		10,040	UK	Improved BP control, increased use of ACEi	High level buy-in generated engagement
CKD interventions aimed at patients	Patient education	Blakeman et al. (2014) [39]	Patient guidebook, telephone guided help from a lay health worker	Booklet and website linking to community resources	436	UK	Improved BP control, increased QALYs	85.7 % uptake rate
		Thomas et al. (2013) [38]	Leaflet, DVD, self-monitoring diary	Single practitioner education and shadowing session	116	UK	Decreased BP	Limited data on level of implementation
		Thomas et al. (2014) [37]	Group education session, leaflet, DVD	Practice training and monthly teleconferences. Patient advisory group	671	UK	Moderate decreases in BP	Patient advisory group involved in design, grant application, delivering education and feedback
	Other	Cottrell et al. (2012) [44]	Mobile phone text messaging BP service		124	UK	No changes in BP, improved prescribing	Many more BP readings

*Abbreviations*: *GP* general practitioner, *eGFR* estimated glomerular filtration rate, *BP* blood pressure, *CKD* chronic kidney disease, *ACEi* angiotensin-converting-enzyme inhibitor, *PTH* parathyroid hormone, *HbA1C* glycated haemoglobin, *QALYs* quality-adjusted life years

### Contexts

We found inconsistencies in the reporting contextual information across the papers included in the review. Many studies did not directly address the issue of ‘context’ from which to make any meaningful inferences. Four papers [23–26] provided very minimal contextual data. Despite variations in the data provided to highlight contextual factors, several key issues were isolated.

The review of the literature identified resource management as a key contextual factor enabling or constraining successful implementation of CKD interventions in primary care. The term ‘resource’ was defined from its broader perspective, encompassing many different types of ‘resource’. These included time [16, 25, 27, 28], finance [16, 29], core and ancillary staff [16, 30], higher-level support [16, 29–32], secondary care services [26, 33], opportunities for benchmarking [16, 34], computer systems [23, 30, 35, 36] and amongst others. CKD interventions were complex and required diverse forms of resources to be available in order for implementation to be fully achieved. All the studies were performed in developed countries with ample resources, including eight studies from the UK, two from other European countries and eight more from North America (see Table 2). However, it was the effective management of resources that enabled mechanisms to facilitate successful implementation. Without effective resource allocation, the lack of key resources prevented mechanisms from making positive outcomes in terms of implementation.

An important issue highlighted by the literature was the change in this context over time, i.e. a shift in resources over time. All eight studies produced in the UK [16, 24, 29, 33, 37–40] were published after 2008, 2 years after CKD was first included in the primary care pay for performance system (the Quality and Outcomes Framework (QOF)) allowing more political priorities and economic resources to be placed on CKD. In addition, different times of the year also affected outcomes, as resources for projects were affected due to other competing priorities such as the QOF pay for performance year-end deadlines, influenza vaccinations, staff changeover, holiday periods and sickness [16, 37]. Furthermore, technological advances such as automated laboratory eGFR reporting allowed more studies to utilise more advanced functions within computer systems and reduced the workload and resources required to diagnose CKD [41]. This allowed many more mechanisms to come into action.

### Mechanisms

**Fig. 2:**
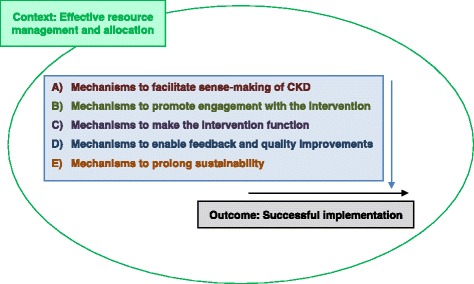
A basic outline of the underlying context and groups of mechanisms that contributed to the outcome of successful implementation of chronic kidney disease (CKD) interventions in primary care

The mechanisms identified from the literature have been grouped using each of the different domains from the NPT framework, in order to correspond with the theoretical foundation that guided generation of data and our analysis (see Fig. 2). All interventions which had features that were successfully implemented had mechanisms leading to a positive outcome of one or more of the four NPT ‘domains’. An additional mechanism related to prolonging sustainability was also identified as important.

**Fig. 3:**
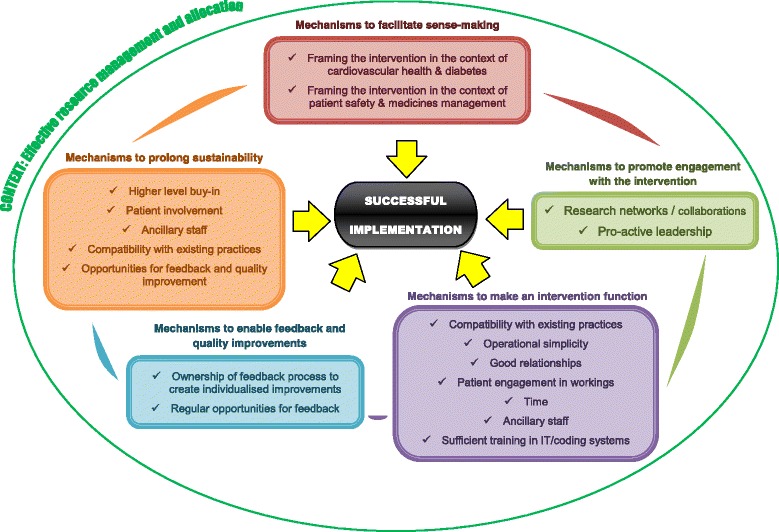
A diagram summarising the mechanisms enabling successful implementation and the overarching context of effective resource management and allocation. The domains from the Normalisation Process Theory (NPT) framework were integrated into key groups of mechanisms, with mechanisms to prolong sustainability as an additional group. Each group of mechanisms also connected to others, having a considerable level of inter-dependence

Figure 3 summarises our findings regarding the numerous mechanisms that contributed to successful implementation, bounded by the context of effective resource management and allocation. As the figure and the proceeding discussion illustrates, there was a considerable level of inter-dependence between the core mechanisms, as predicted by NPT (see also Additional file 4).

### Mechanisms to facilitate sense-making of chronic kidney disease (coherence)

Understanding CKD was an important mechanism and a vital prerequisite for acceptance and subsequent implementation of an intervention. Participants of the intervention needed to understand the importance of CKD as well as the potential benefits of improving its management in primary care. By achieving this, users better engaged with the intervention and were frequently willing to go the extra mile, even if it meant more work. They were also more likely to take part in feedback and quality improvement, as the work was meaningful.

CKD appeared to be a distinct diagnosis where practitioners were reluctant to use it as a ‘label’, with many being unsure of the benefits of disclosing the diagnosis, for fear of causing unintended anxiety [42]. Confidence in managing CKD was lower than for hypertension and diabetes, with practitioners harbouring doubts as to the significance of clinical guideline targets, such as blood pressure [15, 32, 43]. Hence, successful sense-making was more challenging than with many other chronic diseases.

To enable sense-making, the framing of CKD was critical for success. For CKD interventions involving medical professionals, the literature supports framing CKD in the context of cardiovascular health and diabetes [30, 32, 33, 37, 38, 44]. Fox et al. [30] described how clinicians ‘prioritised treatment for diabetes or cardiovascular disease without realising the underlying pathophysiologic link between these diseases and CKD’. Another example of successful framing was in the form of patient safety and medicines management [35, 45]. With an ageing population correlating with a higher prevalence of CKD, patients were frequently subject to polypharmacy [27]. The literature suggested that doctors in primary care had reduced interactions with pharmacists and were supportive of any assistance to enable safe dosing in CKD [45]. In a qualitative review of medication, dosing support software by Erler et al. [35] found that the majority practices used the intervention ‘frequently’ or ‘very often’, with nearly all respondents rating ‘very useful’. Examples of unsuccessful framing included addressing CKD solely in the context of published CKD guidelines [36] and in terms of bone health [36], both of which were not seen as a priority by practitioners. For example, Drawz et al. [36] delivered education on CKD guidelines and how to access a CKD registry, with the primary outcome as parathyroid hormone measurement. However, uptake of the intervention was poor with only 5 out of 37 general practitioners (GPs) accessing the CKD registry.

For CKD interventions aimed at patients, the literature suggests that framing CKD in the context of general well-being and vascular health achieves sense-making [37–39, 44]. Cardiovascular disease is more widely publicised and associating CKD with a common, tangible marker such as blood pressure, helped patients understand the importance of CKD and interventions to improve its management [38, 46]. In a qualitative evaluation of an education intervention including a DVD [38], some patients reported that they particularly ‘took note’ of the information about BP control and found it ‘very interesting’.

### Mechanisms to promote engagement with the intervention (cognitive participation)

Without engagement with the intervention, interventions were not implemented to their full potential, with aspects of the intervention being omitted [34, 38]. Therefore, it was a precondition that had to be fulfilled before work could be performed to enable the intervention to function.

A key intervention feature that facilitated engagement with CKD interventions was the resourcing of research networks or collaborations [16, 23, 27, 30, 34]. Through resourcing these networks, there was greater co-operation, healthy competition and a sense of achievability. In two studies [16, 34] that organised collaborative meetings, individual practices were able to observe the extent to which other practices were taking part and the advantages and barriers to intervention implementation and this facilitated engagement. It also facilitated benchmarking and gave an opportunity to learn from other practices’ feedback and experience, which further enabled engagement and implementation [16, 34]. Through time as part of the network or collaboration, these practices also became more experienced in implementing different interventions and hence were more likely to engage, being more aware of the benefits and challenges of participating.

Furthermore, research networks or collaborations also had good research leadership, with pro-active research teams having visited individual practices to promote the intervention and encourage engagement [16, 34]. In addition, many research networks in North America had clinical staff that were heavily involved in research and therefore acted as ‘champions’ in their own practice to promote engagement and implementation [23, 27, 30]. The literature also suggested that whole practice level engagement was important, with a trial of over 5500 patients revealing that even with the same financial rewards, the intervention was less effective in practices without all-embracing or higher level buy-in [16]. Financial incentives were attractive and successful for creating initial engagement with healthcare professionals willing to do extra work in order to achieve certain requirements. A particular example was the national pay for performance scheme in the UK or Quality and Outcomes Framework (QOF) [29]. However, it emphasised more of a checklist approach and provided no further incentive to achieve better than the level required to achieve financial gain.

### Mechanisms to make an intervention function (collective action)

Drawing on the original model for NPT developed by May et al. [47], known as the Normalisation Process Model (NPM), the mechanism concerning collective action can be split into a four sub-domains: interactional workability, relational integration, skill-set workability and contextual integration. We included these four sub-domains in the reviewing and reporting of the mechanisms, as they focus more on practical work compared to other sub-domains, both on an individual and an organisational level. Further experience in intervention operations helped reduce workload and increase job efficiency [16, 30]. This appeared to be a self-perpetuating mechanism.

#### Operating the intervention and intervention output (interactional workability)

The literature supports the proposition that CKD interventions that were easier to operate were more readily adopted into routine practice [23, 38]. This was especially the case for CKD interventions aimed at patients where ease of use resulted in high levels of uptake of interventions [37–39]. Conversely, for interventions with a computer software component, complicated user interfaces discouraged their usage [35, 36].

The output of the intervention depended on the type of intervention utilised (see Table 2). The intervention type can be separated into two broad contexts: CKD interventions aimed at healthcare professionals and CKD interventions aimed at patients. Within these, they can broadly be categorised into educational, group meetings, multidisciplinary management, computer software and others. We could not identify a link between different types of intervention and the degree of success of implementation. However, it was noted that many of the interventions were multimodal and there was a degree of cross-over between the groups, with many studies providing education sessions to both the intervention and control groups, even when the primary intervention was unrelated.

Many information technology (IT) systems or computerised interventions were designed to reduce manpower and increase data processing speeds in order to reduce workload and time burden [36]. Therefore, they were popular intervention choices with many practices keen for initial buy-in and trialling the systems [23, 30, 35, 36]. Nevertheless, though computerised interventions were viewed as critical factors for successful chronic disease management, they were insufficient for efficient implementation alone [31]. It was also apparent that they had many limitations [23, 36]. When software was slow and disrupted the consultation, the users frequently stopped utilising the intervention [35]. Automatic or passive alerts were frequently ignored or overlooked, proving to be an inferior form of communication compared to direct person-to-person contact [23, 30].

#### Relationships and trust (relational integration)

Good relationships were a key mechanism driving the implementation of CKD interventions. Successful interventions encouraged further relationships between doctors, patients and other healthcare professionals.

##### Relationships between doctors and patients

Interventions which enhanced the doctor-patient relationship were well received both by doctors and patients. In particular, patient-centred interventions, which were theory informed were more successfully implemented, with higher uptake and received positive feedback [28, 33, 37–39]. For example, Blakeman et al. [39] utilised a ‘minimally disruptive medicine’ approach [48] on self-management programmes and received a high uptake of the intervention (85.7 %), with the majority of participants rating the intervention useful.

Another patient-centred intervention was a telehealth programme where patients checked their BP at home using electronic sphygmomanometers and texted their results by mobile phone to the GP or practice nurse [44]. Both practitioners and patients were highly supportive of the flexibility of home BP monitoring, which enabled more frequent readings (daily), but also reduced workload and made it more convenient for patients by reduced clinic appointments [46]. Furthermore, one unintended outcome from the intervention was that patients highly valued the ‘support and companionship’ that was generated by the text message feedback from doctors and nurses about their BP [46]. The healthcare team reported an enhanced doctor to patient rapport, which in turn promoted better management [46].

Moreover, patient engagement in research was viewed as a beneficial influence [37, 46]. This included participatory roles in the setup process and grant applications [37], expert patients to deliver aspects of educational programmes [37] and also focus groups for feedback and improvement [44]. They enhanced the doctor to patient relationship and generated further engagement for both practitioners and patients. They also helped the intervention to function as they were frequently volunteers, and as an extra resource, they contributed to longer sustainability.

##### Relationships between doctors and other healthcare professionals

The literature showed that limited interaction and communication between doctors and other healthcare professionals were barriers to successful implementation. In one study [45], pharmacists indirectly communicated with practitioners by written recommendations to advise medication changes, but only 40 % of recommendations were adopted. This was in contrast to other similar interventions, where medication recommendations achieved rates of over 70 % acceptance through direct communication [49–51].

On the other hand, pro-active multidisciplinary interventions [25, 27, 33] that enhanced relationships between different clinical teams were well received. For different healthcare professionals, this enabled co-operation to reduce workload and improve implementation. In a study by Barrett et al. [25], doctors and nurses worked together to reduce workload by using phones and email rather than the traditional referral system to condense labour. A good relationship also allowed different teams to be more aware of the requirements and barriers to implementation. For example, in the context of CKD, a higher rate of eGFR reporting from a computer decision support system resulted in increased secondary care referrals [26]. Clinicians in secondary care were concerned and required this increase in workload to be resourced before interventions could be fully implemented.

#### Task performance and allocation (skill-set workability)

Another recurring theme from the literature was that time was a critical factor that enhanced implementation and improved performance [16, 25, 27, 28]. Time improved relationships between doctors and patients (relational integration) and allowed more work to be comprehensively carried out at each stage of implementation. Financial incentives were used in one study [16] to resource protected time, whereas another study [28] reduced clinical workload to enable more time to be devoted to intervention operations. Both these strategies greatly improved engagement and increased job performance. In a separate intervention [25], a nurse-led team was given further time and responsibility in managing patients with CKD. With adequate time, healthcare staff embraced the opportunity to manage patients more comprehensively and were even willing to spend their own extra time towards performing related tasks (e.g. driving to collect a forgotten urine sample from a patient’s home) [52].

Conversely, adding work to practices hampered integration and consumed time. Without sufficient time, the intervention was limited in what it could achieve and provided a barrier to intervention uptake. Both doctors and patients reported that time-consuming interventions discouraged their use [35, 38]. ‘External’ project changes and increased administrative workload including excessive feedback work (see ‘Mechanisms to enable feedback and quality improvements’ section) were time-consuming and negative factors for job performance and implementation [37].

Utilising ancillary staff as a strategic resource was crucial to the propagation and sustainability of several interventions [16, 30]. As a separate member of staff from the research team, many tasks that were fundamental to the implementation of the intervention could be allocated to them. Many studies [16, 36] reported the lack of expertise in coding/IT systems, and this formed a barrier to intervention implementation causing problems with labour intensive data collection and inadequacy of data. Even when single training sessions were provided, this issue persisted [36]. In studies where ancillary staff adopted this role and aided practices in either data collection or continuous troubleshooting, implementation proved to be less problematic. In one study [30], ancillary staff also facilitated individual ownership of the intervention and tailoring the intervention to the needs of the clinician by addressing feedback and performing improvement work. They also performed extra tasks such as producing and translating patient information leaflets, which further increased the engagement in the intervention.

#### Compatibility with existing practices (contextual integration)

CKD interventions that were not compatible with existing practices, such as computer systems that were not integrated into the existing practice software, discouraged users to utilise the intervention and created further work (for example, additional data input) that was seen as unconstructive [35, 36]. On the other hand, integrating computer systems with existing software and interventions that operated alongside existing practices were much more popular [23, 27, 33].

In CKD interventions designed for patients as end-users, successful contextual integration was in the form of patients’ everyday lives and convenience. In the telehealth programme [44], there was an increase in compliance of BP readings when patients were able to check their own BP from the convenience of their homes with a high uptake rate of the intervention. Likewise, Blakeman et al. [39] designed their patient intervention specifically to link into existing social and clinical services and reduce disruptions in patients’ schedules. This was associated with successful embedding of the intervention again with a high uptake rate.

### Mechanisms to enable feedback and quality improvements (reflexive monitoring)

An opportunity for feedback and reflection was crucial to encourage sustainability and improvement of an intervention. By improving the intervention, users further engaged and were better able to observe the effects and importance of the intervention.

Ownership of the feedback and quality improvement process by users was important for enhancing implementation [30, 37]. By enabling individualised feedback and quality improvement to occur, patients and clinicians were more willing to engage as they felt they could change the intervention to suit their needs. Positive changes that occurred as a result of quality improvement further encouraged engagement and also made the workings of the intervention more efficient reducing workload [16, 30, 35].

Another mechanism suggested by the literature is the idea that feedback needed to be regular. This was demonstrated by the results of a follow-up study [53], 2 years after the implementation of a clinical decision support system (CDSS) by Fox et al. [30]. Although the results 2 years post intervention were still higher than the original baseline, Wentworth et al. [53] discovered that results of the intervention (including percentage of patients with a diagnosis of CKD, anaemia and BP within range) had decreased after feedback was stopped. This suggests that an element of recurrent feedback or appraisal was required for the optimal effects of an intervention to be sustained.

Two large studies [16, 34] that resourced benchmarking opportunities allowed individual and practice reflection and generated healthy competition and co-operation. This encouraged feedback and enabled individual practices to learn from each other’s successes and mistakes. One particular study [16] further resourced local level quality improvement cycles to good effect, with regular input from improvement facilitators. This assisted engagement and reduced workload.

However, quality improvement (QI) work was not a priority for practices and was an additional task that required further resources and support [16]. In addition, excessive feedback and QI work can even prevent implementation. In a self-management intervention [37], at least 6 out of 19 participating practices were unable to sustain submitting monthly feedback data for 6 months. This was one of the reasons for high dropout rates, with only 13 out of 29 practices completing the study.

### Mechanisms to prolong sustainability

One of the largest challenges facing the incorporation of CKD interventions into everyday practice was sustainability. The literature provided limited insight into whether interventions were successfully incorporated into normal practice. Only six papers [16, 29, 30, 37–39] provided evidence that aspects of the intervention were continued. This was likely due to the recentness of the reviewed studies that may not have had a chance to publish follow-up or evaluative studies. The majority of interventions seemed to be withdrawn after the study. Many of these were pilot studies that were discontinued (at least temporarily) until further resource and funding was available. Hence, larger projects with a larger resource pool appeared to be more sustainable. This was enabled if there was higher level buy-in from governments or larger research networks and collaborations [16, 29–32].

As mentioned above, both patient involvement and ancillary staff had a positive effect on sustainability [16, 30, 37, 44, 46]. Their contributions towards a project as their specific task and as an extra resource appeared to be superior to maintain intervention operation, compared to healthcare professionals whom viewed clinical work as their primary role.

Two key barriers to sustainability were poor contextual integration and a lack of opportunity for feedback and quality improvement [23, 35]. As mentioned in the previous section, feedback needed to be regular in order for the optimal effect of an intervention to be sustained [30, 53].

## Discussion

This rapid realist review aimed to understand factors affecting the implementation of CKD interventions in primary care. Based on our findings, the effective management and allocation of resources (encompassing many different forms) was a key contextual factor that enabled mechanisms to facilitate successful implementation. It was important to take into account that this factor changed over time, which affected how mechanisms worked. The heterogeneity of general practice meant that effective resource management had different requirements in each setting. A recent study examining global challenges of CKD has also highlighted the importance of directed and effective resource allocation [54]. However, we also discovered that there were widespread variations in the reporting of contextual data. This is consistent with previous research examining implementation, which all report discrepancies on how ‘context’ was defined [55–57].

Although the literature did not offer clarity in terms of the importance of one mechanism over another, our review suggests three key intervention features that were particularly important enablers of implementation according to NPT. These were appropriate framing of the intervention, compatibility with existing practices and improvements to create ownership of an intervention. When interventions presented these features, and were supported by adequate resource allocation and management, the core mechanisms functioned effectively and the intervention was successfully implemented.

1. Appropriate framing of the intervention:
  Patients and practitioners are still reported to have a low awareness of CKD [2, 42]. Therefore, to understand the importance of the intervention and the potential benefits of improving CKD management, appropriate framing of the intervention was vital. The UK Department of Health supports the view that work surrounding CKD appears more important when linked into cardiovascular disease and diabetes [58]. Couser et al. [59] also support this view that CKD should be managed and prioritised within the management of other chronic diseases. This approach also appeared to make the workload more amenable and focussed [58].
2. Compatibility with existing practices:
  Both patients and practitioners were reluctant to use interventions that did not fit into everyday practices. This notion of compatibility is emphasised by diffusion of innovations theory and enables a product or idea to become more widespread and better meet users’ requirements [60]. This has also been highlighted as a major factor in previous research into the receiving of new technology and interventions, where compatibility is imperative for end-user acceptance [61].
3. Improvements to create ownership of an intervention:
  Allowing users to interact with the feedback process enabled individualised improvements to the intervention to suit their needs, creating an ‘ownership’ of the intervention and aiding its implementation. Research into translating organisational characteristics from the private to the public sector by Bate et al. [62] has already highlighted the positive effects of ownership and customisable adaptations to systems. Previous evaluations of several national health improvement programmes concluded that it was crucial to generate ownership of an intervention by refining the customisable elements to enable users to further engage with the programmes [63].

### Strengths and limitations

We have established a framework to understand the complex processes surrounding implementation by integrating NPT with realist methodology to describe the individual and collective work of embedding and integrating CKD interventions into a particular context. Our methodology allowed the dissection of each intervention to identify separate components within an intervention that were well implemented and other parts that were not. Previous studies have reported variability in understanding the NPT constructs and dealing with overlapping data that could have affected the final analysis [64, 65]. Allowing for this limitation, NPT was useful in grappling with complex issues and the discussion around how the data fit into each construct allowed a deeper exploration into additional factors and challenged assumptions. NPT formed a pragmatic structure to explore complex factors surrounding implementation including the use of an explicit data sensitisation and categorisation tool. Not only did it enhance the analysis to identify factors that enabled or constrained implementation but also allowed an exploration into the relationships between different mechanisms.

As with any theory, NPT offered the potential to both structure and constrain data. A key tension was the danger of ‘forcing’ data and constructs into the categories delineated by the framework [66, 67]. Our approach adopted the propositions within the NPT and NPM, and assumed the preformed constructs, which were used to categorise our results. Accepting this assumption, the NPT framework allowed further illumination of different types of work concerning implementation at the micro-level and also the relationships between them. Indeed, data could have been missed as analysis was performed through a theoretical lens, as previous studies have noted [68, 69]. However, our approach also identified factors that fell outside the NPT framework. A mechanism that was not explicitly included in the NPT framework for analysis was ‘work to prolong sustainability’. NPT appears to be designed to evaluate factors which might increase sustainability [21]. However, long-term sustainability was a different complex process to initial implementation and was heavily affected by the continual changes in resource management. Unfortunately, data was limited regarding which interventions continued to be used and for how long, and this requires further research.

We acknowledge that a rapid realist review is not a comprehensive search and is not explicitly reproducible, rather it is an iterative and adaptable process guided by testing and refining theories and explanations to produce results most pertinent to practice [20]. Our findings are limited to taking face value acceptance of author’s accounts, working on the assumption that the authors’ understandings were correct. Our synthesis worked mainly with secondary data, and our mechanisms were the third level constructions that were repeatedly tested with the data as part of an iterative process. Another limitation is that we did not have stakeholder engagement as outlined by Saul [20] as part of a rapid realist review. However, the authors of our review team were involved with the development of different CKD interventions and provided access to unpublished data.

A wide range of quantitative and qualitative articles were included in our synthesis, and certain sources provided more data. As all study designs were included, there were differing strengths of evidence and variations in methodological quality. However, excluding study types would limit the amount of data generated to understand processes surrounding implementation. For similar reasons, we opted against the use of a rigid critical appraisal tool, especially with the inclusion of grey literature. Instead, rigour was maintained with frequent discussions regarding the records included and the data extracted by two authors in weekly data sessions. In addition, as consistent with a realist review, in order to enhance trustworthiness, our findings and theories were iteratively tested and retested with the literature.

Our study did not examine effectiveness, but rather how interventions might be implemented to achieve their optimal potential. With this in mind, further research is required to determine the factors that enable CKD interventions to be effective. In addition, there were certainly gaps in evidence within the literature with limited data to perform a cost analysis and also to evaluate prolonged sustainability. Only one study [16] commented on cost and cautioned that their collaborative intervention was resource intensive. It is probable that an optimised intervention designed to enable multiple mechanisms for successful implementation will utilise significant funds and resources. Therefore, cost-analyses of CKD interventions are imperative to identify worthy investments.

### Implications for policy and practice

Healthcare resources have always been limited, whether delivered in a private or public setting [70]. In the current economic climate, effective resource management is of increasing importance. From a UK perspective, despite financial incentives that have improved the management of CKD through the QOF, a pilot report from the national CKD audit showed that only approximately 50 % of patients with stages 3–5 of the disease are being correctly coded on primary care systems [71]. CKD indicators have been removed from the QOF CKD domain, and it is uncertain where the extra resources will come from [72]. The National Kidney Foundation (NKF) did not support these changes, hypothesising that millions of patients could go undetected as a result of this change [73]. Policymakers need to be mindful to compensate for fewer resources available to support CKD management.

AKI has been targeted as a preventable condition by several global and national initiatives [7]. However, none of the 18 primary care CKD interventions that we examined included an outcome measure that explored or included AKI. The International Society of Nephrology’s ‘0by25’ programme [74] aims to prevent all deaths due to untreated AKI by 2025 and NHS England’s ‘Think Kidneys’ Programme has been established [75] to reduce AKI-related morbidity and mortality in hospital and in the community. The impact of the prevention of AKI needs to be incorporated into CKD interventions in future research, which will broaden the scope of kidney disease work to include the interrelated acute and chronic spectrum. This has the potential to increase the importance of CKD for both practitioners and patients and provide a further link between primary and secondary care CKD work for the future.

## Conclusions

This rapid realist review summarises the literature surrounding the implementation of CKD interventions in primary care. Combining NPT with realist methodology allowed an in-depth exploration and helped identify contextual factors and mechanisms that enable and constrain CKD implementation. These factors should be considered to optimise intervention design to improve the management of CKD in primary care. We were unable to draw strong conclusions on long-term sustainability or cost as there was a limited body of evidence, and this requires further research.

## Additional files

Additional file 1: Data extraction tool. (DOC 102 kb)

Additional file 2: Diagram of secondary search process. (DOCX 17 kb)

Additional file 3: Summary table and references for secondary search. (DOCX 204 kb)

Additional file 4: Conceptual framework of the relationships between different mechanisms contributing to the successful implementation of chronic kidney disease interventions in primary care. These mechanisms fell into categories corresponding to the domains of the Normalisation Process Theory (NPT) including: ‘Coherence’ (that is, sense-making work), ‘Cognitive participation’ (engagement work), ‘Collective action’ (operational/functional work), and ‘reflexive monitoring’ (feedback and quality improvement work) [21]. Mechanisms prolonging sustainability was an additional category that appeared to be important. (DOCX 47 kb)

## Abbreviations

ACEi: angiotensin-converting-enzyme inhibitor
AKI: acute kidney injury
BP: blood pressure
CDSS: clinical decision support system
CKD: chronic kidney disease
eGFR: estimated glomerular filtration rate
GP: general practitioner
HbA1C: glycated haemoglobin
IT: information technology
NKF: National Kidney Foundation
NPM: Normalisation Process Model
NPT: Normalisation Process Theory
PTH: parathyroid hormone
QALYs: quality-adjusted life years
QOF: Quality and Outcomes Framework
