# Supplementary material for: Understanding the implementation of interventions to improve the management of chronic kidney disease in primary care: a rapid realist review
Source: Implement Sci. 2016 Apr 4;11:47. doi: 10.1186/s13012-016-0413-7 (PMC4820872; doi:10.1186/s13012-016-0413-7)
Supplement: Supplementary file 1 — Data extraction tool. (DOC 102 kb) [file 13012_2016_413_MOESM1_ESM.doc]

| Title |  |
| --- | --- |
| Date published |  |
| Author |  |
| Design |  |
| Sample size |  |
| Specific demographics |  |
| Setting |  |
| Costs and grants |  |
| Date of study |  |
| Intervention |  |
| Nature (focus of intervention) |  |
| Primary outcomes |  |
| Secondary outcomes |  |
| Other outcomes |  |
| Results |  |
| Major Limitations |  |
| Conclusions |  |
|  |  |
| Underpinning theories |  |
| Other comments |  |

| Coherence  (i.e., meaning and sense-making by participants) | Is the intervention easy to describe? |  |
| --- | --- | --- |
|  | Is it clearly distinct from other interventions? |  |
|  | Does it have a clear purpose for all relevant participants? |  |
|  | Do participants have a shared sense of its purpose? |  |
|  | What benefits will the intervention bring and to whom? |  |
|  | Are these benefits likely to be valued by potential participants? |  |
|  | Will it fit with the overall goals and activity of the organisation? |  |
|  | | |
| Cognitive participation  (i.e., commitment and  engagement by participants) | Are target user groups likely to think it is a good idea? |  |
|  | Will they see the point of the intervention easily? |  |
|  | Will they be prepared to invest time, energy and work in it? |  |
|  | | |
| Collective Action  (i.e., the work participants do to make the intervention function) | How will the intervention affect the work of user groups? |  |
|  | Will it promote or impede their work? |  |
|  | What effect will it have on consultations? |  |
|  | Will staff require extensive training before they can use it? |  |
|  | How compatible is it with existing work practices? |  |
|  | What impact will it have on division of labour, resources, power, and responsibility between different professional groups? |  |
|  | | |
| Reflexive Monitoring  (i.e., participants reflect on or appraise the intervention) | How are users likely to perceive the intervention once it has been in use for a while? |  |
|  | Is it likely to be perceived as advantageous for patients or staff? |  |
|  | Will it be clear what effects the intervention has had? |  |
|  | Can users/staff contribute feedback about the intervention once it is in use? |  |
|  | Can the intervention be adapted/ improved on the basis of experience? |  |
