# Supplementary material for: Understanding the implementation of interventions to improve the management of chronic kidney disease in primary care: a rapid realist review
Source: Implement Sci. 2016 Apr 4;11:47. doi: 10.1186/s13012-016-0413-7 (PMC4820872; doi:10.1186/s13012-016-0413-7)
Supplement: Supplementary file 2 — Diagram of secondary search process. (DOCX 17 kb) [file 13012_2016_413_MOESM2_ESM.docx]

**Studies identified from the primary search**(n = 18)

**Total number of records screened in secondary search**(n = 4902)

**Total records generated from reference lists**(n = 706)

**Records returned via Google for author and research groups (each capped at 100)**(n = 3600)

**Records returned via pubmed for authors**(n = 596)

**Screening & de-duplication**

**Secondary records included to inform primary studies**(n = 147)
