# Supplementary material for: Understanding the implementation of interventions to improve the management of chronic kidney disease in primary care: a rapid realist review
Source: Implement Sci. 2016 Apr 4;11:47. doi: 10.1186/s13012-016-0413-7 (PMC4820872; doi:10.1186/s13012-016-0413-7)
Supplement: Supplementary file 3 — Summary table and references for secondary search. (DOCX 204 kb) [file 13012_2016_413_MOESM3_ESM.docx]

| **Author (year)** | **Records returned via reference list** | **Records returned via Pubmed for author** | **Number of records returned via Google for author & research group [each capped at 100]** | **Number of other secondary records included to inform each primary paper $** | **References for each record included** |
| --- | --- | --- | --- | --- | --- |
| Abdel Kader et al (2011) [1] | 57 | 17 | 200 | 16 | [11, 15, 19-32] |
| Akbari et al (2004) [2] | 56 | 50 | 200 | 8 | [21, 33-39] |
| Barrett et al (2011) [3] | 33 | 43 | 200 | 5 | [15, 40-43] |
| Bayliss et al (2011) [4] | 35 | 41 | 200 | 4 | [44-47] |
| Blakeman et al (2014) [5] | 62 | 21 | 200 | 12 | [48-59] |
| Cortes-Sanabria et al. (2008) [6] | 39 | 26 | 200 | 5 | [60-64] |
| Cottrell et al (2012) [7] | 19 | 23 | 200 | 5 | [65-69] |
| De Lusignan et al (2013) [8] | 74 | 140 | 200 | 15 | [70-84] |
| Drawz et al (2012) [9] | 28 | 27 | 200 | 4 | [1, 11, 85, 86] |
| Erler et al (2012) [10] | 51 | 16 | 200 | 12 | [87-98] |
| Fox et al (2008) [11] | 27 | 48 | 200 | 9 | [99-107] |
| Humphreys et al (2012) [12] | 23 | 41 | 200 | 7 | [108-114] |
| Karunaratne et al (2013) [13] | 33 | 17 | 200 | 15 | [115-128] |
| Patel et al (2005) [14] | 21 | 3 | 200 | 4 | [129-131] |
| Richards et al (2008) [15] | 49 | 9 | 200 | 13 | [45, 86, 132-142] |
| Scherpbier et al (2013) [16] | 37 | 6 | 200 | 4 | [143-146] |
| Thomas et al (2013) [17] | 44 | 34 | 200 | 6 | [147-152] |
| Thomas et al (2014) [18] | 18 | 34 | 200 | 3 | [152-154] |

$Note that the number of records is not representative of the quality of data extracted for each primary paper. Rather, some with fewer sources had better descriptive papers to inform the primary study

# References

1. Abdel-Kader K, Fischer GS, Li J, Moore CG, Hess R, Unruh ML: **Automated clinical reminders for primary care providers in the care of CKD: A small cluster-randomized controlled trial**. *American Journal of Kidney Diseases* 2011, **58**(6):894-902.

2. Akbari A, Swedko PJ, Clark HD, Hogg W, Lemelin J, Magner P, Moore L, Ooi D: **Detection of chronic kidney disease with laboratory reporting of estimated glomerular filtration rate and an educational program**. *Archives of Internal Medicine* 2004, **164**(16):1788-1792.

3. Barrett BJ, Garg AX, Goeree R, Levin A, Molzahn A, Rigatto C, Singer J, Soltys G, Soroka S, Ayers D *et al*: **A nurse-coordinated model of care versus usual care for stage 3/4 chronic kidney disease in the community: a randomized controlled trial**. *Clin J Am Soc Nephrol* 2011, **6**(6):1241-1247.

4. Bayliss EA, Bhardwaja B, Ross C, Beck A, Lanese DM: **Multidisciplinary team care may slow the rate of decline in renal function**. *Clinical Journal of the American Society of Nephrology* 2011, **6**(4):704-710.

5. Blakeman T, Blickem C, Kennedy A, Reeves D, Bower P, Gaffney H, Gardner C, Lee V, Jariwala P, Dawson S *et al*: **Effect of information and telephone-guided access to community support for people with chronic kidney disease: Randomised controlled trial**. *PLoS ONE* 2014, **9**(10).

6. Cortes-Sanabria L, Cabrera-Pivaral CE, Cueto-Manzano AM, Rojas-Campos E, Barragan G, Hernandez-Anaya M, Martinez-Ramirez HR: **Improving care of patients with diabetes and CKD: a pilot study for a cluster-randomized trial**. *American Journal of Kidney Diseases* 2008, **51**(5):777-788.

7. Cottrell E, Chambers R, O'Connell P: **Using simple telehealth in primary care to reduce blood pressure: A service evaluation**. *BMJ Open* 2012, **2**(6).

8. De Lusignana S, Gallagher H, Jones S, Chan T, Van Vlymen J, Tahir A, Thomas N, Jain N, Dmitrieva O, Rafi I *et al*: **Audit-based education lowers systolic blood pressure in chronic kidney disease: The Quality Improvement in CKD (QICKD) trial results**. *Kidney International* 2013, **84**(3):609-620.

9. Drawz PE, Miller RT, Singh S, Watts B, Kern E: **Impact of a chronic kidney disease registry and provider education on guideline adherence--a cluster randomized controlled trial**. *BMC medical informatics and decision making* 2012, **12**:62.

10. Erler A, Beyer M, Petersen JJ, Saal K, Rath T, Rochon J, Haefeli WE, Gerlach FM: **How to improve drug dosing for patients with renal impairment in primary care - a cluster-randomized controlled trial**. *BMC family practice* 2012, **13**:91.

11. Fox CH, Swanson A, Kahn LS, Glaser K, Murray BM: **Improving chronic kidney disease care in primary care practices: an upstate New York practice-based research network (UNYNET) study**. *J Am Board Fam Med* 2008, **21**(6):522-530.

12. Humphreys J, Harvey G, Coleiro M, Butler B, Barclay A, Gwozdziewicz M, O'Donoghue D, Hegarty J: **A collaborative project to improve identification and management of patients with chronic kidney disease in a primary care setting in Greater Manchester**. *BMJ Quality and Safety* 2012, **21**(8):700-708.

13. Karunaratne K, Stevens P, Irving J, Hobbs H, Kilbride H, Kingston R, Farmer C: **The impact of pay for performance on the control of blood pressure in people with chronic kidney disease stage 3-5**. *Nephrology Dialysis Transplantation* 2013, **28**(8):2107-2116.

14. Patel HR, Pruchnicki MC, Hall LE: **Assessment for chronic kidney disease service in high-risk patients at community health clinics**. *Annals of Pharmacotherapy* 2005, **39**(1):22-27.

15. Richards N, Harris K, Whitfield M, O'Donoghue D, Lewis R, Mansell M, Thomas S, Townend J, Eames M, Marcelli D: **Primary care-based disease management of chronic kidney disease (CKD), based on estimated glomerular filtration rate (eGFR) reporting, improves patient outcomes**. *Nephrol Dial Transplant* 2008, **23**(2):549-555.

16. Scherpbier-de Haan ND, Vervoort GMM, Weel C, Braspenning JCC, Mulder J, Wetzels JFM, De Grauw WJC: **Effect of shared care on blood pressure in patients with chronic kidney disease: A cluster randomised controlled trial**. *British Journal of General Practice* 2013, **63**(617):e798-e806.

17. Thomas N, Bryar R: **An evaluation of a self-management package for people with diabetes at risk of chronic kidney disease**. *Primary health care research & development* 2013, **14**(3):270-280.

18. Thomas N, Gallagher H, Jain N: **A quality improvement project to improve the effectiveness and patient-centredness of management of people with mild-to-moderate kidney disease in primary care**. *BMJ Quality Improvement Reports* 2014, **3**(1).

19. Balas EA, Weingarten S, Garb CT, Blumenthal D, Boren SA, Brown GD: **Improving preventive care by prompting physicians**. *Arch Intern Med* 2000, **160**(3):301-308.

20. Weiner M, Callahan CM, Tierney WM, Overhage JM, Mamlin B, Dexter PR, McDonald CJ: **Using information technology to improve the health care of older adults**. *Ann Intern Med* 2003, **139**(5 Pt 2):430-436.

21. Bero LA, Grilli R, Grimshaw JM, Harvey E, Oxman AD, Thomson MA: **Closing the gap between research and practice: an overview of systematic reviews of interventions to promote the implementation of research findings. The Cochrane Effective Practice and Organization of Care Review Group**. *Bmj* 1998, **317**(7156):465-468.

22. Smith WR: **Evidence for the effectiveness of techniques To change physician behavior**. *Chest* 2000, **118**(2 Suppl):8s-17s.

23. Ash JS, Sittig DF, Campbell EM, Guappone KP, Dykstra RH: **Some unintended consequences of clinical decision support systems**. *AMIA Annu Symp Proc* 2007:26-30.

24. Isaac T, Weissman JS, Davis RB, Massagli M, Cyrulik A, Sands DZ, Weingart SN: **Overrides of medication alerts in ambulatory care**. *Arch Intern Med* 2009, **169**(3):305-311.

25. Shah NR, Seger AC, Seger DL, Fiskio JM, Kuperman GJ, Blumenfeld B, Recklet EG, Bates DW, Gandhi TK: **Improving acceptance of computerized prescribing alerts in ambulatory care**. *J Am Med Inform Assoc* 2006, **13**(1):5-11.

26. Nies J, Colombet I, Degoulet P, Durieux P: **Determinants of success for computerized clinical decision support systems integrated in CPOE systems: a systematic review**. *AMIA Annu Symp Proc* 2006:594-598.

27. Kawamoto K, Houlihan CA, Balas EA, Lobach DF: **Improving clinical practice using clinical decision support systems: a systematic review of trials to identify features critical to success**. *Bmj* 2005, **330**(7494):765.

28. Garg AX, Adhikari NK, McDonald H, Rosas-Arellano MP, Devereaux PJ, Beyene J, Sam J, Haynes RB: **Effects of computerized clinical decision support systems on practitioner performance and patient outcomes: a systematic review**. *Jama* 2005, **293**(10):1223-1238.

29. McCoy AB, Waitman LR, Gadd CS, Danciu I, Smith JP, Lewis JB, Schildcrout JS, Peterson JF: **A computerized provider order entry intervention for medication safety during acute kidney injury: a quality improvement report**. *Am J Kidney Dis* 2010, **56**(5):832-841.

30. Wipfli R, Lovis C: **Alerts in clinical information systems: building frameworks and prototypes**. *Stud Health Technol Inform* 2010, **155**:163-169.

31. Shahinian VB, Saran R: **The role of primary care in the management of the chronic kidney disease population**. *Adv Chronic Kidney Dis* 2010, **17**(3):246-253.

32. Rutkowski M, Mann W, Derose S, Selevan D, Pascual N, Diesto J, Crooks P: **Implementing KDOQI CKD definition and staging guidelines in Southern California Kaiser Permanente**. *Am J Kidney Dis* 2009, **53**(3 Suppl 3):S86-99.

33. Noble E, Johnson DW, Gray N, Hollett P, Hawley CM, Campbell SB, Mudge DW, Isbel NM: **The impact of automated eGFR reporting and education on nephrology service referrals**. *Nephrol Dial Transplant* 2008, **23**(12):3845-3850.

34. Grimshaw JM, Russell IT: **Effect of clinical guidelines on medical practice: a systematic review of rigorous evaluations**. *Lancet* 1993, **342**(8883):1317-1322.

35. Davis DA, Thomson MA, Oxman AD, Haynes RB: **Changing physician performance. A systematic review of the effect of continuing medical education strategies**. *JAMA* 1995, **274**(9):700-705.

36. Tamblyn R, Battista R: **Changing clinical practice: Which interventions work?** *Journal of Continuing Education in the Health Professions* 1993, **13**(4):273-288.

37. Wensing M, Grol R: **Single and combined strategies for implementing changes in primary care: a literature review**. *Int J Qual Health Care* 1994, **6**(2):115-132.

38. Wensing M, van der Weijden T, Grol R: **Implementing guidelines and innovations in general practice: which interventions are effective?** *Br J Gen Pract* 1998, **48**(427):991-997.

39. Solberg LI, Brekke ML, Kottke TE: **Are physicians less likely to recommend preventive services to low-SES patients?** *Prev Med* 1997, **26**(3):350-357.

40. Van Zuilen AD, Wetzels JF, Bots ML, Van Blankestijn PJ: **MASTERPLAN: study of the role of nurse practitioners in a multifactorial intervention to reduce cardiovascular risk in chronic kidney disease patients**. *J Nephrol* 2008, **21**(3):261-267.

41. Molzahn AE, Hibbert MP, Gaudet D, Starzomski R, Barrett B, Morgan J: **Managing chronic kidney disease in a nurse-run, physician-monitored clinic: the CanPREVENT experience**. *Can J Nurs Res* 2008, **40**(3):96-112.

42. Katon WJ, Lin EH, Von Korff M, Ciechanowski P, Ludman EJ, Young B, Peterson D, Rutter CM, McGregor M, McCulloch D: **Collaborative care for patients with depression and chronic illnesses**. *N Engl J Med* 2010, **363**(27):2611-2620.

43. Rettig R, Vargas R, Norris K, Nissenson A: **Chronic kidney disease - A quiet revolution in nephrology. Six case studies.** In: *RAND Technical report.* 2010.

44. Ghossein C, Serrano A, Rammohan M, Batlle D: **The role of comprehensive renal clinic in chronic kidney disease stabilization and management: The Northwestern experience**. *Semin Nephrol* 2002, **22**(6):526-532.

45. Harris LE, Luft FC, Rudy DW, Kesterson JG, Tierney WM: **Effects of multidisciplinary case management in patients with chronic renal insufficiency**. *Am J Med* 1998, **105**(6):464-471.

46. Hemmelgarn BR, Manns BJ, Zhang J, Tonelli M, Klarenbach S, Walsh M, Culleton BF: **Association between multidisciplinary care and survival for elderly patients with chronic kidney disease**. *J Am Soc Nephrol* 2007, **18**(3):993-999.

47. Curtis BM, Ravani P, Malberti F, Kennett F, Taylor PA, Djurdjev O, Levin A: **The short- and long-term impact of multi-disciplinary clinics in addition to standard nephrology care on patient outcomes**. *Nephrol Dial Transplant* 2005, **20**(1):147-154.

48. Blakeman T, Protheroe J, Chew-Graham C, Rogers A, Kennedy A: **Understanding the management of early-stage chronic kidney disease in primary care: a qualitative study**. *Br J Gen Pract* 2012, **62**(597):e233-242.

49. May C, Montori VM, Mair FS: **We need minimally disruptive medicine**. *Bmj* 2009, **339**:b2803.

50. Blickem C, Kennedy A, Vassilev I, Morris R, Brooks H, Jariwala P, Blakeman T, Rogers A: **Linking people with long-term health conditions to healthy community activities: development of Patient-Led Assessment for Network Support (PLANS)**. *Health Expect* 2013, **16**(3):e48-59.

51. Osborne RH, Elsworth GR, Whitfield K: **The Health Education Impact Questionnaire (heiQ): an outcomes and evaluation measure for patient education and self-management interventions for people with chronic conditions**. *Patient Educ Couns* 2007, **66**(2):192-201.

52. Toobert DJ, Hampson SE, Glasgow RE: **The summary of diabetes self-care activities measure: results from 7 studies and a revised scale**. *Diabetes Care* 2000, **23**(7):943-950.

53. Zigmond AS, Snaith RP: **The hospital anxiety and depression scale**. *Acta Psychiatr Scand* 1983, **67**(6):361-370.

54. Broadbent E, Petrie KJ, Main J, Weinman J: **The brief illness perception questionnaire**. *J Psychosom Res* 2006, **60**(6):631-637.

55. Russell DW: **UCLA Loneliness Scale (Version 3): reliability, validity, and factor structure**. *J Pers Assess* 1996, **66**(1):20-40.

56. Morisky DE, Green LW, Levine DM: **Concurrent and predictive validity of a self-reported measure of medication adherence**. *Med Care* 1986, **24**(1):67-74.

57. Kennedy A, Reeves D, Bower P, Lee V, Middleton E, Richardson G, Gardner C, Gately C, Rogers A: **The effectiveness and cost effectiveness of a national lay-led self care support programme for patients with long-term conditions: a pragmatic randomised controlled trial**. *J Epidemiol Community Health* 2007, **61**(3):254-261.

58. Kind P: **The EuroQoL instrument: an index of health-related quality of life**. *Quality of life and pharmacoeconomics in clinical trials* 1996, **2**:191-201.

59. Bajekal M, Purdon S: **Social capital and social exclusion: development of a condensed module for the Health Survey for England**. *London: National Centre for Social Research* 2001.

60. Snyder S, Pendergraph B: **Detection and evaluation of chronic kidney disease**. *Am Fam Physician* 2005, **72**(9):1723-1732.

61. Cabrera-Pivaral CE, Chavez SA, Gonzalez-Reyes HF, Cortes-Sanabria L: **[Clinical aptitude of family doctors in the management of patients with diabetes type two with initial nephropathy]**. *Rev Invest Clin* 2005, **57**(5):685-690.

62. Viniegra VL: **La crítica: aptitud olvidada por la educación**. *México: Instituto Mexicano del Seguro Social* 2000:1-25.

63. Bowen JL: **Educational Strategies to Promote Clinical Diagnostic Reasoning**. *New England Journal of Medicine* 2006, **355**(21):2217-2225.

64. Brown N, Doshi M: **Assessing professional and clinical competence: the way forward**. *Advances in Psychiatric Treatment* 2006, **12**(2):81-89.

65. Cottrell E, McMillan K, Chambers R: **A cross-sectional survey and service evaluation of simple telehealth in primary care: what do patients think?** *BMJ Open* 2012, **2**(6).

66. Clark M, Goodwin N, Network WSDA: **Sustaining innovation in telehealth and telecare**: WSD Action Network, King's Fund; 2010.

67. Jones MI, Greenfield SM, Bray EP, Baral-Grant S, Hobbs FD, Holder R, Little P, Mant J, Virdee SK, Williams B *et al*: **Patients' experiences of self-monitoring blood pressure and self-titration of medication: the TASMINH2 trial qualitative study**. *Br J Gen Pract* 2012, **62**(595):e135-142.

68. Bostock Y, Hanley J, McGown D, Pinnock H, Padfield P, McKinstry B: **The acceptability to patients and professionals of remote blood pressure monitoring using mobile phones**. *Primary Health Care Research & Development* 2009, **10**(04):299-308.

69. McManus RJ, Mant J, Bray EP, Holder R, Jones MI, Greenfield S, Kaambwa B, Banting M, Bryan S, Little P *et al*: **Telemonitoring and self-management in the control of hypertension (TASMINH2): a randomised controlled trial**. *Lancet* 2010, **376**(9736):163-172.

70. de Lusignan S, Gallagher H, Chan T, Thomas N, van Vlymen J, Nation M, Jain N, Tahir A, du Bois E, Crinson I *et al*: **The QICKD study protocol: a cluster randomised trial to compare quality improvement interventions to lower systolic BP in chronic kidney disease (CKD) in primary care**. *Implementation Science : IS* 2009, **4**:39-39.

71. De Lusignan S: **An educational intervention; involving feedback of routinely collected computer data, to improve cardiovascular disease management in UK primary care**. *Methods of Information in Medicine* 2007, **46**(1):57-62.

72. Stevens PE, O'Donoghue DJ, De Lusignan S, Van Vlymen J, Klebe B, Middleton R, Hague N, New J, Farmer CKT: **Chronic kidney disease management in the United Kingdom: NEOERICA project results**. *Kidney International* 2007, **72**(1):92-99.

73. de Lusignan S, van Weel C: **The use of routinely collected computer data for research in primary care: opportunities and challenges**. *Fam Pract* 2006, **23**(2):253-263.

74. de Lusignan S, Hague N, Brown A, Majeed A: **An educational intervention to improve data recording in the management of ischaemic heart disease in primary care**. *J Public Health (Oxf)* 2004, **26**(1):34-37.

75. de Lusignan S, Belsey J, Hague N, Dhoul N, van Vlymen J: **Audit-based education to reduce suboptimal management of cholesterol in primary care: a before and after study**. *J Public Health (Oxf)* 2006, **28**(4):361-369.

76. De Goeij MCM, Rotmans JI: **Audit-based education: A potentially effective program for improving guideline achievement in CKD patients**. *Kidney International* 2013, **84**(3):436-438.

77. Rafi I, Chowdhury S, Chan T, Jubber I, Tahir M, de Lusignan S: **Improving the management of people with a family history of breast cancer in primary care: before and after study of audit-based education**. *BMC Fam Pract* 2013, **14**:105.

78. Forsetlund L, Bjorndal A, Rashidian A, Jamtvedt G, O'Brien MA, Wolf F, Davis D, Odgaard-Jensen J, Oxman AD: **Continuing education meetings and workshops: effects on professional practice and health care outcomes**. *Cochrane Database Syst Rev* 2009(2):Cd003030.

79. Jamtvedt G, Young JM, Kristoffersen DT, O'Brien MA, Oxman AD: **Audit and feedback: effects on professional practice and health care outcomes**. *Cochrane Database Syst Rev* 2006(2):Cd000259.

80. Gardner B, Whittington C, McAteer J, Eccles MP, Michie S: **Using theory to synthesise evidence from behaviour change interventions: the example of audit and feedback**. *Soc Sci Med* 2010, **70**(10):1618-1625.

81. Hysong SJ: **Meta-analysis: audit and feedback features impact effectiveness on care quality**. *Med Care* 2009, **47**(3):356-363.

82. Hulscher ME, Wensing M, van Der Weijden T, Grol R: **Interventions to implement prevention in primary care**. *Cochrane Database Syst Rev* 2001(1):Cd000362.

83. Mitchell E, Sullivan F, Grimshaw JM, Donnan PT, Watt G: **Improving management of hypertension in general practice: a randomised controlled trial of feedback derived from electronic patient data**. *Br J Gen Pract* 2005, **55**(511):94-101.

84. Soumerai SB, Avorn J: **Principles of educational outreach ('academic detailing') to improve clinical decision making**. *Jama* 1990, **263**(4):549-556.

85. Green CJ, Fortin P, Maclure M, Macgregor A, Robinson S: **Information system support as a critical success factor for chronic disease management: Necessary but not sufficient**. *Int J Med Inform* 2006, **75**(12):818-828.

86. Wagner EH, Austin BT, Davis C, Hindmarsh M, Schaefer J, Bonomi A: **Improving chronic illness care: translating evidence into action**. *Health Aff (Millwood)* 2001, **20**(6):64-78.

87. Terrell KM, Perkins AJ, Hui SL, Callahan CM, Dexter PR, Miller DK: **Computerized decision support for medication dosing in renal insufficiency: a randomized, controlled trial**. *Ann Emerg Med* 2010, **56**(6):623-629.

88. Schoen C, Osborn R, Doty MM, Squires D, Peugh J, Applebaum S: **A survey of primary care physicians in eleven countries, 2009: perspectives on care, costs, and experiences**. *Health Aff (Millwood)* 2009, **28**(6):w1171-1183.

89. Kaushal R, Shojania KG, Bates DW: **Effects of computerized physician order entry and clinical decision support systems on medication safety: a systematic review**. *Arch Intern Med* 2003, **163**(12):1409-1416.

90. Kuperman GJ, Bobb A, Payne TH, Avery AJ, Gandhi TK, Burns G, Classen DC, Bates DW: **Medication-related clinical decision support in computerized provider order entry systems: a review**. *J Am Med Inform Assoc* 2007, **14**(1):29-40.

91. Shamliyan TA, Duval S, Du J, Kane RL: **Just what the doctor ordered. Review of the evidence of the impact of computerized physician order entry system on medication errors**. *Health Serv Res* 2008, **43**(1 Pt 1):32-53.

92. Wolfstadt JI, Gurwitz JH, Field TS, Lee M, Kalkar S, Wu W, Rochon PA: **The effect of computerized physician order entry with clinical decision support on the rates of adverse drug events: a systematic review**. *J Gen Intern Med* 2008, **23**(4):451-458.

93. Bryan C, Boren SA: **The use and effectiveness of electronic clinical decision support tools in the ambulatory/primary care setting: a systematic review of the literature**. *Inform Prim Care* 2008, **16**(2):79-91.

94. Varonen H, Kortteisto T, Kaila M: **What may help or hinder the implementation of computerized decision support systems (CDSSs): a focus group study with physicians**. *Fam Pract* 2008, **25**(3):162-167.

95. Short D, Frischer M, Bashford J: **Barriers to the adoption of computerised decision support systems in general practice consultations: a qualitative study of GPs' perspectives**. *Int J Med Inform* 2004, **73**(4):357-362.

96. Eslami S, Abu-Hanna A, de Keizer NF: **Evaluation of outpatient computerized physician medication order entry systems: a systematic review**. *J Am Med Inform Assoc* 2007, **14**(4):400-406.

97. Leifermann M, Gerlach FM, Beyer M, Petersen JI, Erler A: **Family practitioner's evaluation of an electronic tool for dose adjustment in patients with chronic kidney disease - A qualitative study**. *Zeitschrift fur Allgemeinmedizin* 2010, **86**(4):146-151.

98. Ash JS, Fournier L, Stavri PZ, Dykstra RH: **Principles for a successful computerized physician order entry implementation**. In: *AMIA: 2003*; 2003.

99. Fox CH, Brooks A, Zayas LE, McClellan W, Murray B: **Primary care physicians' knowledge and practice patterns in the treatment of chronic kidney disease: An Upstate New York Practice-based Research Network (UNYNET) study**. *Journal of the American Board of Family Medicine* 2006, **19**(1):54-61.

100. Wentworth AL, Fox CH, Kahn LS, Glaser K, Cadzow R: **Two years after a quality improvement intervention for chronic kidney disease care in a primary care office**. *American Journal of Medical Quality* 2011, **26**(3):200-205.

101. Glasgow RE, Vogt TM, Boles SM: **Evaluating the public health impact of health promotion interventions: the RE-AIM framework**. *Am J Public Health* 1999, **89**(9):1322-1327.

102. Nagykaldi Z, Mold JW, Aspy CB: **Practice facilitators: a review of the literature**. *Fam Med* 2005, **37**(8):581-588.

103. Nagykaldi Z, Mold JW, Robinson A, Niebauer L, Ford A: **Practice facilitators and practice-based research networks**. *J Am Board Fam Med* 2006, **19**(5):506-510.

104. Soman S, Zasuwa G, Yee J: **Automation, decision support, and expert systems in nephrology**. *Adv Chronic Kidney Dis* 2008, **15**(1):42-55.

105. Denekamp Y: **Clinical decision support systems for addressing information needs of physicians**. *Isr Med Assoc J* 2007, **9**(11):771-776.

106. O’Brien MA, Rogers S, Jamtvedt G, Oxman AD, Odgaard-Jensen J, Kristoffersen DT, Forsetlund L, Bainbridge D, Freemantle N, Davis D: **Educational outreach visits: effects on professional practice and health care outcomes**. *Cochrane database syst rev* 2007, **4**(4).

107. Hartig JR, Allison J: **Physician performance improvement: an overview of methodologies**. *Clin Exp Rheumatol* 2007, **25**(6 Suppl 47):50-54.

108. Harvey G, Fitzgerald L, Fielden S, McBride A, Waterman H, Bamford D, Kislov R, Boaden R: **The NIHR collaboration for leadership in applied health research and care (CLAHRC) for greater manchester: combining empirical, theoretical and experiential evidence to design and evaluate a large-scale implementation strategy**. *Implementation Science* 2011, **6**(1):1-12.

109. Kitson AL, Rycroft-Malone J, Harvey G, McCormack B, Seers K, Titchen A: **Evaluating the successful implementation of evidence into practice using the PARiHS framework: theoretical and practical challenges**. *Implement Sci* 2008, **3**(1):1.

110. Ferlie E, Dopson S, Fitzgerald L, Locock L: **RENEWING POLICY TO SUPPORT EVIDENCE‐BASED HEALTH CARE**. *Public Administration* 2009, **87**(4):837-852.

111. McCormack B, Kitson A, Harvey G, Rycroft‐Malone J, Titchen A, Seers K: **Getting evidence into practice: the meaning ofcontext'**. *Journal of advanced nursing* 2002, **38**(1):94-104.

112. J O, Bate P, Cleary P, Cretin S, Gustafson D, McInnes K, McLeod H, Molfenter T, Plsek P, Robert G *et al*: **Quality collaboratives: lessons from research**. *Qual Saf Health Care* 2002, **11**(4):345-351.

113. Wilson T, Berwick DM, Cleary PD: **What do collaborative improvement projects do? Experience from seven countries**. *Jt Comm J Qual Saf* 2003, **29**(2):85-93.

114. Hogg W, Baskerville N, Lemelin J: **Cost savings associated with improving appropriate and reducing inappropriate preventive care: cost-consequences analysis**. *BMC Health Serv Res* 2005, **5**(1):20.

115. Health and Social Care information Centre. **Quality and Outcomes Framework** [<http://www.hscic.gov.uk/qof>] Accessed 7 Apr 2015.

116. Carey IM, Nightingale CM, DeWilde S, Harris T, Whincup PH, Cook DG: **Blood pressure recording bias during a period when the Quality and Outcomes Framework was introduced**. *J Hum Hypertens* 2009, **23**(11):764-770.

117. Serumaga B, Ross-Degnan D, Avery AJ, Elliott RA, Majumdar SR, Zhang F, Soumerai SB: **Effect of pay for performance on the management and outcomes of hypertension in the United Kingdom: interrupted time series study**. *Bmj* 2011, **342**:d108.

118. Stevens PE, Farmer CKT, de Lusignan S: **Effect of pay for performance on hypertension in the United kingdom**. *Am J Kidney Dis* 2011, **58**(4):508-511.

119. Millett C, Bottle A, Ng A, Curcin V, Molokhia M, Saxena S, Majeed A: **Pay for perfomance and the quality of diabetes management in individuals with and without co-morbid medical conditions**. *J R Soc Med* 2009, **102**(9):369-377.

120. Langdown C, Peckham S: **The use of financial incentives to help improve health outcomes: is the quality and outcomes framework fit for purpose? A systematic review**. *Journal of Public Health* 2013.

121. Fleetcroft R, Cookson R: **Do the incentive payments in the new NHS contract for primary care reflect likely population health gains?** *Journal of Health Services Research & Policy* 2006, **11**(1):27-31.

122. Doran T, Kontopantelis E, Valderas JM, Campbell S, Roland M, Salisbury C, Reeves D: **Effect of financial incentives on incentivised and non-incentivised clinical activities: longitudinal analysis of data from the UK Quality and Outcomes Framework**. *BMJ* 2011, **342**.

123. Dixon A, Khachatryan A, Wallace A, Peckham S, Boyce T, Gillam S: **Impact of Quality and Outcomes Framework on health inequalities**. *London: The King's Fund* 2011.

124. Lester H, Matharu T, Mohammed MA, Lester D, Foskett-Tharby R: **Implementation of pay for performance in primary care: a qualitative study 8 years after introduction**. *British Journal of General Practice* 2013, **63**(611):e408-e415.

125. McGregor W, Jabareen H, O'Donnell CA, Mercer SW, Watt GC: **Impact of the 2004 GMS contract on practice nurses: a qualitative study**. *Br J Gen Pract* 2008, **58**(555):711-719.

126. Guthrie B, McLean G, Sutton M: **Workload and reward in the Quality and Outcomes Framework of the 2004 general practice contract**. *Br J Gen Pract* 2006, **56**(532):836-841.

127. Ashworth M, Armstrong D: **The relationship between general practice characteristics and quality of care: a national survey of quality indicators used in the UK Quality and Outcomes Framework, 2004–5**. *BMC family practice* 2006, **7**(1):1.

128. Checkland K, Harrison S: **The impact of the QOF on practice organisation and service delivery**. *The Quality and Outcomes Framework: QOF-Transforming General Practice* 2010:93-107.

129. Chisholm MA, Vollenweider LJ, Mulloy LL, Jagadeesan M, Wade WE, DiPiro JT: **Direct patient care services provided by a pharmacist on a multidisciplinary renal transplant team**. *Am J Health Syst Pharm* 2000, **57**(21):1994-1996.

130. Galt KA: **Cost avoidance, acceptance, and outcomes associated with a pharmacotherapy consult clinic in a Veterans Affairs Medical Center**. *Pharmacotherapy* 1998, **18**(5):1103-1111.

131. Rubio-Valera M, Jové A, Hughes CM, Guillen-Solà M, Rovira M, Fernández A: **Factors affecting collaboration between general practitioners and community pharmacists: a qualitative study**. *BMC Health Services Research* 2012, **12**(1):1-10.

132. Chen RA, Scott S, Mattern WD, Mohini R, Nissenson AR: **The case for disease management in chronic kidney disease**. *Dis Manag* 2006, **9**(2):86-92.

133. Wrone EM, Hornberger J: **Evaluating the consequences of multidisciplinary case management for patients with chronic renal failure**. In: *Am J Med. Volume 105*, edn. United states; 1998: 546-548.

134. Nissenson AR, Collins AJ, Dickmeyer J, Litchfield T, Mattern W, McMahill CN, Muhlbaier L, Nielsen J, Owen WF, Jr., Pereira BJ *et al*: **Evaluation of disease-state management of dialysis patients**. *Am J Kidney Dis* 2001, **37**(5):938-944.

135. Bodenheimer T, Wagner EH, Grumbach K: **Improving primary care for patients with chronic illness: the chronic care model, Part 2**. *Jama* 2002, **288**(15):1909-1914.

136. Black DA: **Case management for elderly people in the community**. In: *Bmj. Volume 334*, edn. England; 2007: 3-4.

137. Gravelle H, Dusheiko M, Sheaff R, Sargent P, Boaden R, Pickard S, Parker S, Roland M: **Impact of case management (Evercare) on frail elderly patients: controlled before and after analysis of quantitative outcome data**. *Bmj* 2007, **334**(7583):31.

138. Sheaff R, Boaden R, Sargent P, Pickard S, Gravelle H, Parker S, Roland M: **Impacts of case management for frail elderly people: a qualitative study**. *J Health Serv Res Policy* 2009, **14**(2):88-95.

139. Sargent P, Pickard S, Sheaff R, Boaden R: **Patient and carer perceptions of case management for long-term conditions**. *Health Soc Care Community* 2007, **15**(6):511-519.

140. Schmidt RJ, Domico JR, Sorkin MI, Hobbs G: **Early referral and its impact on emergent first dialyses, health care costs, and outcome**. *Am J Kidney Dis* 1998, **32**(2):278-283.

141. Winkelmayer WC, Schneeweiss S, Mogun H, Patrick AR, Avorn J, Solomon DH: **Identification of individuals with CKD from Medicare claims data: a validation study**. *Am J Kidney Dis* 2005, **46**(2):225-232.

142. Anand S, Nissenson AR: **Utilizing a disease management approach to improve ESRD patient outcomes**. *Semin Dial* 2002, **15**(1):38-40.

143. Black C, Sharma P, Scotland G, McCullough K, McGurn D, Robertson L, Fluck N, MacLeod A, McNamee P, Prescott G *et al*: **Early referral strategies for management of people with markers of renal disease: a systematic review of the evidence of clinical effectiveness, cost-effectiveness and economic analysis**. *Health Technol Assess* 2010, **14**(21):1-184.

144. Dean J: **Organising care for people with diabetes and renal disease**. *J Ren Care* 2012, **38 Suppl 1**:23-29.

145. van Hateren KJ, Drion I, Kleefstra N, Groenier KH, Houweling ST, van der Meer K, Bilo HJ: **A prospective observational study of quality of diabetes care in a shared care setting: trends and age differences (ZODIAC-19)**. *BMJ Open* 2012, **2**(4).

146. Jones C, Roderick P, Harris S, Rogerson M: **An evaluation of a shared primary and secondary care nephrology service for managing patients with moderate to advanced CKD**. *American Journal of Kidney Diseases* 2006, **47**(1):103-114.

147. Coulter A, Ellins J: **Patient-focused interventions: a review of the evidence**: Health Foundation London; 2006.

148. Warsi A, Wang PS, LaValley MP, Avorn J, Solomon DH: **Self-management education programs in chronic disease: a systematic review and methodological critique of the literature**. *Arch Intern Med* 2004, **164**(15):1641-1649.

149. Glasgow RE, Fisher L, Skaff M, Mullan J, Toobert DJ: **Problem solving and diabetes self-management: investigation in a large, multiracial sample**. *Diabetes Care* 2007, **30**(1):33-37.

150. Curtin RB, Walters BA, Schatell D, Pennell P, Wise M, Klicko K: **Self-efficacy and self-management behaviors in patients with chronic kidney disease**. *Adv Chronic Kidney Dis* 2008, **15**(2):191-205.

151. Li T, Wu HM, Wang F, Huang CQ, Yang M, Dong BR, Liu GJ: **Education programmes for people with diabetic kidney disease**. *Cochrane Database Syst Rev* 2011(6):Cd007374.

152. Thomas NM, Bryar R, Makanjuola D: **Development of a self-management package for people with diabetes at risk of chronic kidney disease (CKD)**. *Journal of Renal Care* 2008, **34**(3):151-158.

153. Thomas N, Watson S, Rafi I, Barnes K, George E, Harris K: **Can a care bundle approach be used to implement best practice for the management of diabetes and chronic kidney disease in primary care?** *Diabetic Medicine* 2010, **1)**:187.

154. Kidney Research UK. **A Package of Innovation for managing kidney disease in primary care** [https://www.kidneyresearchuk.org/health-information/resources/package-of-innovation] Accessed 7 Apr 2015.
