# Supplementary material for: Understanding the implementation of interventions to improve the management of chronic kidney disease in primary care: a rapid realist review
Source: Implement Sci. 2016 Apr 4;11:47. doi: 10.1186/s13012-016-0413-7 (PMC4820872; doi:10.1186/s13012-016-0413-7)
Supplement: Supplementary file 4 — Conceptual framework of the relationships between different mechanisms contributing to the successful implementation of chronic kidney disease interventions in primary care. These mechanisms fell into categories corresponding to the domains of the Normalisation Process Theory (NPT) including: ‘Coherence’ (that is, sense-making work), ‘Cognitive participation’ (engagement work), ‘Collective action’ (operational/functional work), and ‘reflexive monitoring’ (feedback and quality improvement work) [21]. Mechanisms prolonging sustainability was an additional category that appeared to be important. (DOCX 47 kb) [file 13012_2016_413_MOESM4_ESM.docx]

**Reduced workload Increased efficiency**

**Meaningful work**

**Improvements**

**Using an intervention to its potential**

- Research networks/collaborations
- Pro-active leadership

**Successful engagement**

- Framing the intervention in the context of vascular health and diabetes
- Framing the intervention in the context of patient safety and medicines management

**Successful sense making**

- Ownership of feedback process to create individualised improvements
- Regular opportunities for feedback

**Successful feedback and quality improvements**

- Compatibility with existing practices
- Operational simplicity
- Good relationships
- Patient engagement in workings
- Time
- Ancillary staff
- Sufficient training in IT/coding systems

**Successful functioning of the intervention**

- Higher level buy-in
- Patient involvement
- Ancillary staff
- Compatibility with existing practices
- Opportunities for feedback and quality improvement

**Prolonged sustainability**

**Familiarisation and practice**
